# Excess Mortality among Physicians and Dentists during COVID-19 in Italy: A Cross-Sectional Study Related to a High-Risk Territory

**DOI:** 10.3390/healthcare10091684

**Published:** 2022-09-04

**Authors:** Saturnino Marco Lupi, Claudia Todaro, Domenico Camassa, Silvana Rizzo, Stefano Storelli, Ruggero Rodriguez y Baena

**Affiliations:** 1 Department of Clinical Surgical, Diagnostic and Pediatric Sciences, University of Pavia, P.le Golgi 2, 27100 Pavia, Italy; 2 Department of Biomedical, Surgical and Dental Sciences, University of Milan, Via Beldiletto 1/3, 20142 Milan, Italy

**Keywords:** COVID-19, SARS-CoV-2, mortality rate, healthcare workers

## Abstract

Background: Many studies previously reported epidemiological data on mortality due to COVID-19 among health workers. All these studies included a partial sample of the population with a substantial selection bias. The present study evaluates the trend of mortality among physicians and dentists operating in an area considered to be at high risk during the COVID-19 pandemic. Methods: Data relating to all physicians and dentists registered in the province of Pavia (Italy), a sample consisting of 5454 doctors in 2020 was analyzed. The mortality rates obtained were compared with those related to the 5-year period preceding the pandemic and with those related to the general population. Results: In the area considered, a mortality rate of 0.83% (+69% compared to 2015–2019) was observed in the entire sample in 2020 and 0.43% (−11% compared to 2015–2019) in 2021; among physicians, there was a mortality rate of 0.76% (+53% compared to 2015-2019) in 2020 and 0.35% (−29% compared to 2015–2019) in 2021; for dentists, there was a mortality rate of 1.27% (+185% compared to 2015–2019) in 2020 and 1.01% (+127% compared to 2015–2019) in 2021. Conclusions: These data report the global impact of the SARS-CoV-2 pandemic on physicians and dentists in a high-risk territory. In 2020, a significant increase in the mortality rate compared to the previous 5 years was observed for both physicians and dentists; in 2021, a significant increase in the mortality rate was observed only for dentists. These data are also significant in evaluating the impact of vaccination on physicians and dentists and indicate that dentists were among the professions most at risk during the pandemic.

## 1. Introduction

At the end of 2019, a new severe acute respiratory syndrome-related coronavirus (SARS-CoV-2) was reported for the first time in Wuhan city, Hubei Province, China, causing a pandemic from which there has not yet been a complete recovery [1,2]. The disease linked to SARS-CoV-2 infection was called COVID-19 [3]. Italy is one of the countries with the greatest increase in mortality during this epidemic [4]. Various explanations were proposed for this high death rate: the older age distribution in Italy, the broad definition of COVID-19-related deaths extended to all deceased patients who tested positive for SARS-CoV-2 via real-time polymerase chain reaction (RT-PCR), independently from pre-existing diseases, and the testing strategy which prioritized testing patients with more severe clinical symptoms who were suspected of having COVID-19 and required hospitalization [5,6]. In Italy, COVID-19 had a different diffusion according to the locality, with the highest mortality in northern Italy and in particular in Lombardy [4,7,8]. This difference has not yet been fully explained, although the presence of retirement homes and, as a protective factor, the number of doctors were considered as factors associated with the spread of COVID-19 in the territory [9]. The severity of the epidemic made it necessary to adopt emergency measures [10]. The spread of the virus was modified by different factors, such as lockdowns, vaccination of the population and the appearance of viral variants capable of infecting even vaccinated people [11].

During the COVID-19 pandemic, multiple variants of SARS-CoV-2 appeared with different patterns of transmission and disease severity [12]. One of the first variants of concern (VOCs), alpha (B.1.1.7), was initially detected in England in November 2020 [13]. The delta variant, initially identified in India in December 2020, showed a higher risk of hospitalization and emergency care attendance than the alpha variant [13,14]. The delta variant became widespread from April 2021 and was then almost completely replaced by the omicron variant between December 2021 and January 2022 [15]. The omicron variant was first reported to WHO from South Africa on 24 November 2021 and became predominant at the end of January 2022 [15,16]. A large number of studies indicated that clinical severity of the omicron variant is much lower than the delta, with a strong reduction in the risk of hospitalization and death [17,18,19,20,21,22,23,24].

A determining factor in the evolution of the epidemic was the availability of vaccines. The COVID-19 vaccines were shown to be effective in reducing infectious risk approximately two weeks after the first dose [25]. This effect must be taken into account when assessing the temporal course of the epidemic. In Italy, the vaccination campaign began in early January 2021, when recipients were health sector workers, nursing home residents, people over 80 years of age, policemen, teachers, and frail people. From April 2021, the vaccination campaign was gradually extended to the entire population. This vaccination campaign very effectively reduced mortality and hospitalization [26]. Despite the proven effectiveness in reducing the absolute number of cases and the number of severe cases, the possibility of SARS-CoV-2 infection was also well demonstrated in fully vaccinated subjects [27]. The vaccines demonstrated a moderate reduction in protection against symptomatic infection for the omicron variant; however, the administration of the booster dose allowed one to maintain a high level of protection against hospitalization and death [28,29].

The data on mortality from COVID-19 is limited by the fact that different definitions have been used in different countries and even in the same country [30]. Furthermore, especially early in the outbreak when tests were only available in hospitals, many patients who died outside of a hospital but with clinical signs of COVID-19 had not been diagnosed [5,8]. Excess mortality is a general indicator that measures the increase in deaths compared to a previous period. It allows us to overcome the problem of COVID-19 reporting and diagnosis variability among the various countries and the problem of classification of the cause of death. Assuming that the incidence of death from other causes remains unchanged, the excess mortality makes it possible to identify deaths directly or indirectly due to COVID-19 and returns a global measure of the impact of the pandemic; furthermore, the excess mortality could allow more valid international comparisons [31,32,33,34]. However, being a general indicator, excess mortality cannot analyze the components that determined the variation [31]. Therefore, it also includes the deaths of those patients who, despite needing complex treatment for chronic diseases, had their treatment postponed because they were not urgent cases or they themselves preferred not to go to hospitals for fear of being infected, and worsening their health condition [8,9,35].

Among the different job categories, health workers play a particularly delicate role during a pandemic [36]. An increase in mortality was reported among physicians and dentists and Italy reached the highest rank for the number of deceased physicians and dentists [37,38]. Furthermore, dentists ranked second in the death rate after only general practitioners and emergency room doctors [37]. An increase in mortality among physicians was reported in a previous meta-analysis [39]. However, this meta-analysis included cross-sectional, case-series and retrospective studies regarding the spread of COVID-19 among healthcare professionals; these studies were characterized by providing a precise indication over time or by evaluating the type of symptoms and possibly the mortality rate in a group of people affected by COVID-19; however, it does not provide data on the spread of COVID-19 in a complete sample of health workers in a territory. Certain data is not easy to obtain, firstly, because diagnostic tests were initially available only in the hospital setting, and therefore many deaths have not even been correlated with COVID-19 [9]. Secondly, the classification of the cause of death from COVID-19 has changed over time: initially all deaths in people affected by SARS-CoV-2 were considered to be caused by COVID-19; later, SARS-CoV-2 was considered a cause of death only if the patient died of complications related to it. Among the risk factors for the severe form of COVID-19 can be listed advanced age and male sex, the medical specialty exercised, the lack of adequate training in the management of affected patients, the absence of adequate personal protective equipment which occurred during the first phase of the epidemic, and the belonging to certain ethnic groups [40]. Dentists can be particularly vulnerable to the spread of SARS-CoV-2 [37,40]. In fact, during dental treatment the patient must remove the mask for long periods, the distance between doctor and patient is necessarily close, and droplets are also produced through the use of water-cooled rotating instruments, which contain the blood and saliva of the patient and can spread to the operators [41]. The virus concentration in the pharyngeal, throat and saliva is very high and remains even after the symptoms have disappeared [42]. Among the procedures that expose to greater transmission risk are ultrasonic scaling, highspeed air-rotor, oral surgery, slow-speed handpiece, air-water syringe, air-polishing, prophylaxis, and hand-scaling [41]. In the general population, lockdown, mask use and hand washing with disinfectant solutions were effectively adopted as epidemic containment measures [10,43]. In a survey conducted between the end of March and the beginning of April 2020, 54% of healthcare givers reported inadequate personal protective equipment [44]. In the dental field, precautionary procedures have been implemented to reduce the risk of SARS-CoV-2 contagion [45].

In Italy, the National Institute of Statistics (ISTAT) has been in charge of collecting epidemiological data concerning the COVID-19 epidemic. The data collected include the number of infections and deaths, on a territorial basis up to the minimum municipal level. This organization does not collect data based on profession and has not collected data on the mortality of doctors; consequently, raw data on mortality among physicians and dentists in each territory are not available.

In Italy, physicians and dentists must be registered in a register organized on a provincial basis in order to practice. This register is promptly updated on the cancellation of members due to death, and it publishes the updates via a bulletin. Furthermore, physicians and dentists can be qualified in the register, and therefore exercise the profession, as physicians, dentists, or both. The latter possibility is actually reserved for those doctors who enrolled in the medical course before 1985. Although it is possible that a physician-dentist has the double qualification to practice both medicine and dentistry, these colleagues mainly practice dentistry but they want to keep open the possibility of practicing medicine and therefore keep the double qualification. Conversely, it would be highly unusual for a doctor of any other type, such as a gynecologist or an ophthalmologist, enrolled before 1985, to retain the dual qualification to reserve the right to practice dentistry without practicing it. Therefore, those enrolled with the double qualification must be born in 1966 at the latest and therefore, in 2020, be at least 54 years old.

To our knowledge, no studies have been published so far that have given a comprehensive account of the impact of COVID-19 on the mortality of healthcare workers, and specifically of physicians and dentists.

The purpose of this study is to define mortality in the entire population of physicians and dentists during the COVID-19 epidemic in the years 2020 and 2021 in the province of Pavia, Italy, an area considered to be at high risk for the spread of COVID-19. Furthermore, this study compared mortality between doctors and the general population residing in the same territory.

## 2. Materials and Methods

### 2.1. Participants and Procedure

In this study, the effects of the COVID-19 epidemic were evaluated using the mortality rate because it was considered more reliable than the cause of death [31,32,33,34]. To evaluate the variation in mortality in physicians and dentists linked to the COVID-19 epidemic, the province of Pavia was considered. This is because the province is located in Lombardy, a region in northern Italy in which the epidemic has had particularly serious effects. This province has over 500,000 inhabitants spread over an area of just under 3000 km^2^ [46]. At the beginning of 2020, the total number of registered physicians and dentists was over 5000. The trend of the mortality during COVID-19 epidemic was assessed in the years 2020 and 2021, for which complete data are available relating to the general population and to physicians and dentists. To make a comparison, the mortality data of the previous 5 years (2015–2019) were used, relating both to the general population and to physicians and dentists in the area. To evaluate the trend of the COVID-19 epidemic at the level of the province of Pavia in the population, the mortality data were obtained from the registers of the Italian civil protection and ISTAT [47,48]. Mortality data relative to physicians and dentists were obtained from the provincial register of doctors. Data relating to those enrolled in the register of physicians and dentists were considered in whole and divided according to the qualification, i.e., as physicians, dentists, and physicians-dentists. Furthermore, since physicians-dentists are substantially composed of medical graduates who practice dentistry and are over the age of 54, and instead those enrolled only in the register of dentists are mostly composed of dentists with a degree in dentistry and under the age of 54, the two groups were also merged to increase significance and to be able to make a more correct comparison with the group of physicians only.

### 2.2. Measures

Mortality was expressed in relation to 100 subjects, i.e., as a percentage.

The mortality rate (MR%) was calculated with the following formula when a comparison was made between members of the order:

(1) $$\mathrm{MR}\%=\frac{M_{1}}{M_{0}}\times \;k$$

where:

M_1_ = mortality in 2020 or 2021
M_0_ = mean mortality in 2015–2019
k: multiplicative constant used (100).

For comparison between the mortality of the population and that of those enrolled in the register of physicians and dentists, the mortality rate was adjusted on the basis of the composition of those enrolled in the register of physicians and dentists, grouped by age groups of 5 years (standardized mortality rate, SMR%) as follows:

(2) $$\mathrm{SMR}\%=\frac{\mathrm{Observed}}{\mathrm{Attended}}=\frac{n}{\sum_{i}R_{i}\times P_{i}}\times k$$

where:

Observed: observed mortality
Attended: expected mortality
n: number of deaths among doctors
Ri: mortality rate in the reference population of the age group -i
Pi: number of the population under observation in the population in the age group -i
k: multiplicative constant used (100).

### 2.3. Statistical Analysis

The specific values relating to a single year do not present measures of dispersion. The data relating to the average of the years 2015–2019 are presented as the mean ± standard deviation. The MR% and the SMR% are presented with the respective 95% confidence interval for the evaluation of significance. The probability of having the number of deaths observed in 2020 and 2021, given the average of the period 2015–2019, was also evaluated with the Poisson distribution; *p* < 0.05 was considered significant.

To further clarify the categories observed, refer to Table 1.

This study was reported according to the Strengthening the Reporting of Observational studies in Epidemiology (STROBE) statement [49]. 

## 3. Results

**Figure 1:**
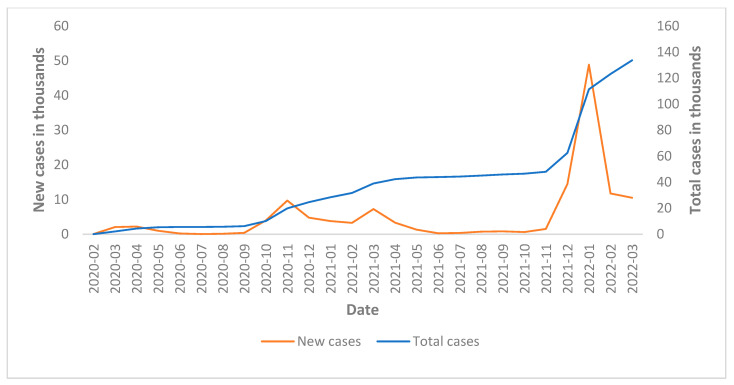
Trend of SARS-CoV-2 infections in the province of Pavia. Data from Italian Civil Protection—Contagion Rates [47].

The trend of COVID-19 infections in Pavia province between the onset of the pandemic and the end of 2021 is shown in Figure 1. There are peaks of infections in March and November 2020, March 2021 and December 2021/January 2022.

**Figure 2:**
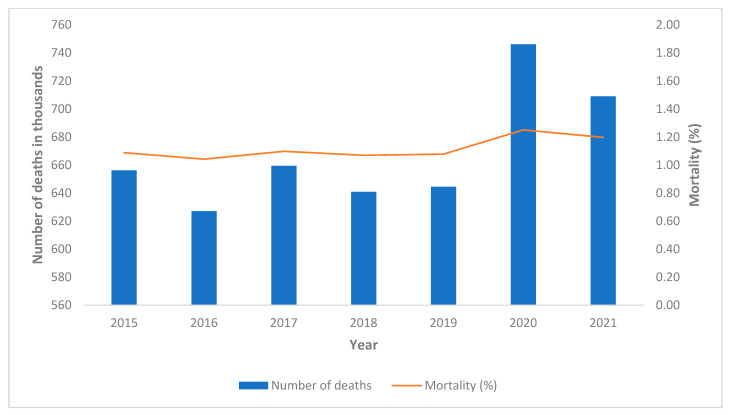
Number of deaths and mortality rate in the Italian population (a).

**Figure 3:**
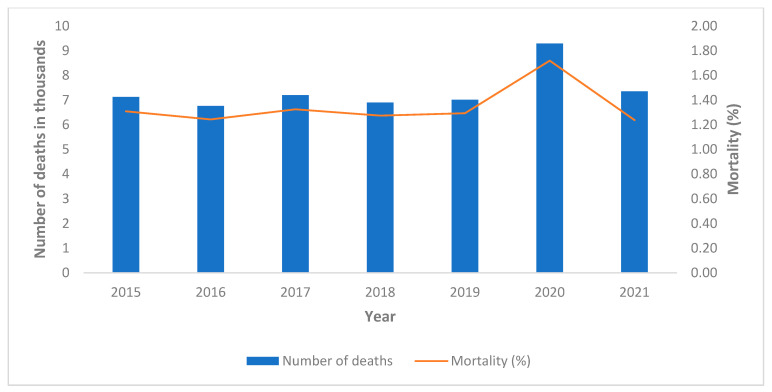
Number of deaths and mortality rate in the population of the province of Pavia (b).

**Figure 4:**
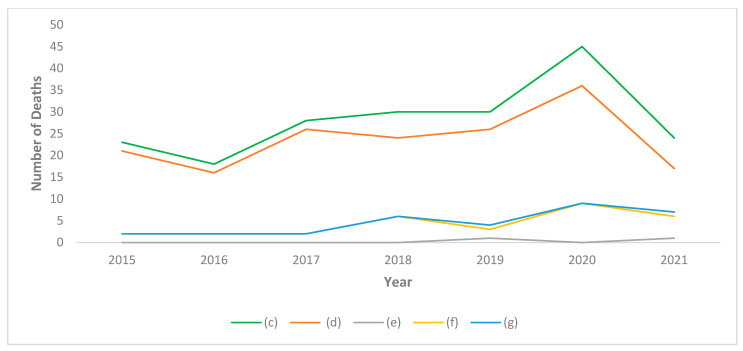
Number of deaths among physicians and dentists in the province of Pavia. (c): All Doctors, (d): Physicians, (e): Dentists, (f): Physicians-Dentists, (g): All Dentists.

The demographic data relating to the Italian population, to the population of the province of Pavia, and to the physicians and dentists are shown in Table 2 and in Figure 2, Figure 3 and Figure 4.

While in Italy the mortality rate in 2020 was 1.25% with an increase of 16.37% compared to the previous 5 years, in the province of Pavia the mortality rate in the same year was 1.72% with an increase of 33.43% compared to the previous year (Figure 2 and Figure 3).

Mortality of all the physicians and dentists (c) went from 0.49% in the 5 years preceding the epidemic, to 0.83% in 2020 and then dropped to 0.43% in 2021 (Figure 4). Population data cannot be directly compared with those of physicians and dentists because the groups are made up of a population of different ages.

The data, relating to the number of deaths among physicians and dentists, shows a peak in the number of deaths relative to 2020, and in particular to April 2020 (data not shown). In absolute terms, the highest number of deaths was observed among physicians (d), followed by physicians-dentists (f) (Figure 4).

**Figure 5:**
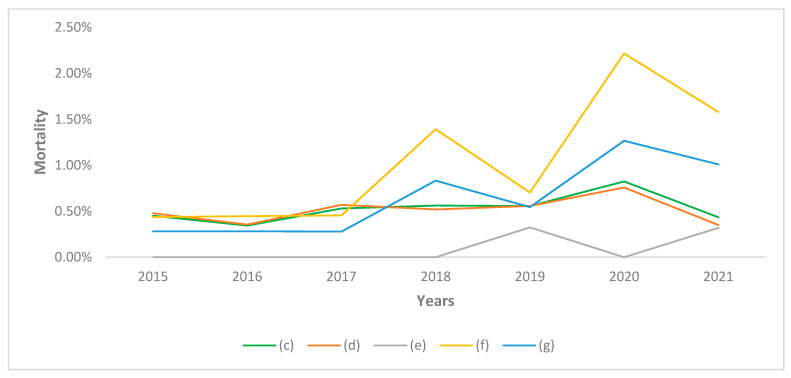
Mortality rate among physicians and dentists in the province of Pavia. (c): All Doctors, (d): Physicians, (e): Dentists, (f): Physicians-Dentists, (g): All Dentists.

Moving on to analyze the mortality rate, a peak was observed in 2020 in all groups of doctors (c,d,f,g) with the exception of dentists (e) (Figure 5). In particular, the highest mortality figure was observed among physicians-dentists (f) both in 2020 and in 2021. Since physicians-dentists (f) are mainly composed of older subjects, and dentists (e) of younger subjects, the most representative data are those relating to dental practitioners as a whole (g). These data show that dentists (g) had a higher mortality rate than physicians in 2020 and 2021.

**Figure 6:**
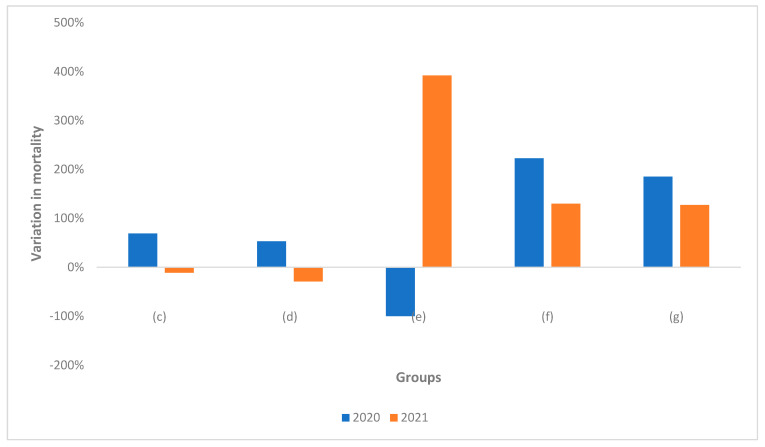
Variation in mortality among physicians and dentists. (c): All Doctors, (d): Physicians, (e): Dentists, (f): Physicians-Dentists, (g): All Dentists.

Compared to the previous 5-year period, the variation in mortality is shown in Figure 6.

The change in mortality compared to 2015–2019 had a very important increase in 2020 in all categories (c,d,f,g) with the exception of dentists (e); in 2021, on the other hand, a decrease in mortality was observed compared to 2015–2019 for all doctors as a whole (c) due to the statistical weight of physicians (d), for whom a decrease in mortality was observed; vice versa, an increase in mortality was also observed in 2021 for all dental practitioners (e,f,g).

The comparison between the mortality data of 2020 and 2021 and the previous 5 years is shown in Table 3.

From the analysis of the mortality indices for 2020, the following can be stated:

- Mortality increased significantly by 53%, 225% and 68% among physicians (d), physician-dentists (f) and total (c), respectively.
- Considering the total number of dentists (g), mortality increased significantly by 175%.

From the analysis of the mortality indices relating to 2021, the following can be stated:

- Mortality increased significantly by 131% among dentists (f).
- Considering the total number of dentists (g), mortality increased significantly by 127%.

Moreover, the mortality of the doctors was compared to that of the resident population.

To do this, it was necessary to calculate the SMR by standardizing it with respect to the general population. In fact, mortality data related to the general population also include subjects of ages that are either too low or too high. Therefore, the mortality indices were balanced with respect to the composition of the members of the order divided by age groups of 5 years.

The results of this analysis are reported in Table 4.

From this analysis it can be deduced that doctors (c, d, f, g) had a significantly lower mortality than the local population with the same composition by age group for all the years considered. Dentists (g) are an exception for whom, however, the data must be taken with caution due to the low number of events.

## 4. Discussion

This study reports the real mortality data of a sample of 5454 doctors and dentists who carried out their activity in one of the territories with one of the highest rates of diffusion and severity of COVID-19.

The peak of infections observed in the province of Pavia in March 2020 corresponds to the onset of the pandemic; the peak in November of the same year could correspond to the alpha variant; the peak observed in March 2021 could correspond to the appearance of the delta variant, and finally, the peak at the end of 2021 and early 2022 could correspond to the omicron variant [15].

In the province of Pavia there was an increase in mortality in the general population of approximately double compared to the national territory. Therefore, it is correct to state that the area of afference of the physicians and dentists included in this study had one of the highest rates of diffusion and severity of COVID-19. The excess mortality could also have been influenced by the overload of the healthcare system, [36,50] which could have led to a reduction in the level of care for both patients with COVID-19 and those with other acute or chronic diseases [9,51,52,53].

A recent meta-analysis reported a mortality rate of 1.5% in a sample of 119,883 healthcare professionals, [39] therefore much higher than that found in this study. This discrepancy can be easily explained because the meta-analysis included many studies in which 100% of the patients were infected with SARS-CoV-2 and, therefore, that finding is not comparable with the results of the present study.

From the mortality data, it is possible to state that in 2020 doctors and dentists (c) had an increase in mortality of approximately double compared to the general reference population (68.83% vs. 33.43%). In 2021, on the other hand, there was a decrease in mortality among doctors and dentists (c) compared to the 5-year period preceding the pandemic, while a slight increase was still observed in the general population. These data may indicate the severity of the spread of the pandemic in the medical profession in 2020; for 2021, the decrease in mortality can probably be explained by the early and complete administration of vaccines among doctors, but also by an increase in the availability of PPE, or by other factors. It is possible to say with certainty that in 2020 in all groups of doctors, except for group (e), a significant increase in mortality was observed compared to the previous 5 years. Furthermore, this significant difference in mortality was also observed in 2021 for dentists (groups f and g).

Furthermore, dentistry practitioners (g) had a different mortality pattern from physicians (d) with mortality rates and mortality increases much higher than physicians in both 2020 and 2021. The particularly high mortality increase among dentistry practitioners (g) can be explained by the high risk due to the particular methods of work, i.e., close distances, the necessary absence of a mask for the patient, and prolonged contact times.

The estimates of dental staff reported a mortality risk related to COVID-19 of 0.008% [54]; the results of the present study do not confirm this estimate and, indeed, find an increase in mortality almost 100 times higher.

Another study reported a 0.06% increase in the death rate among doctors caused by COVID-19 [55]. In this study the difference in mortality observed with respect to the previous years is 0.34%. The differences in the results may have different explanations: first of all, that study was based on the data of all Italian doctors, and therefore the data relating to areas with a low prevalence of COVID-19 may have moderate the results. Secondly, the data from that study are based on COVID-19-related deaths, and so there may have been underestimation linked to a misdiagnosis.

In confirmation of the previous literature [39], from the analysis of the data it is possible to state that physicians and dentists in the province of Pavia had a lower mortality rate than the population of the same territory in the 5 years preceding the pandemic; this can be explained by a better quality of life, understood as involving less exposure to risk factors, but also with a greater predisposition to early diagnosis. Even during the two-year period of the pandemic, the mortality rate of physicians and dentists was lower than that of the general population; however, it has increased much more than that of the general population. This finding has several implications. First, the persistence of a basic risk of death, not related to COVID-19, lower than the general population; then, medical and epidemiological training that could have increased awareness of correct hygienic procedures; finally, in 2021, the early and complete coverage of vaccination with respect to the general population [26,56].

Confirming the previous literature [40], dentists were particularly affected by COVID-19, and dentists had a higher mortality rate than doctors (Figure 5).

Evidently, the data relating to dentists (e) must be considered with caution because the deaths observed are extremely rare and therefore difficult to treat statistically. For this reason, the group of dentists (e) has been merged with that of physicians-dentists (f) to provide a greater representation of dentistry operators. In this way, the group of physicians (d) and that of dentists (g) are made more homogeneous according to age. It can be observed that mortality in the dentist group (g) is lower than that of the physicians-dentists group (f). In fact, physicians-dentists have a higher average age than dentists alone (e). Nonetheless, it can be observed that the mortality rate is significantly higher among those who practiced dentistry (g) compared to physicians (d) in both 2020 and 2021.

Although a significant peak in mortality increases was also observed in 2020 among physicians (d), the data show that in the following year mortality returned to being within the limits of the 5-year period preceding the COVID-19 pandemic; in contrast, mortality among dentists increased in 2020 and in 2021.

Furthermore, mortality among physicians-dentists (f) and among total dentists (g) was higher than that among physicians (d).

Taking into account the excess mortality, for all doctors (c), an excess of mortality of 18 health workers was observed for 2020. To understand how much the pandemic has affected most dentists (g) as a whole, they have suffered a third of the excess deaths while they represent only 13% of the total number of members of the order; from another point of view, among dentists (g) half of the excess deaths of doctors (d) have been observed, although they are only 15% of them.

As regards 2021, even among physicians (d) a negative excess of mortality was observed, i.e., the number of deaths observed was lower than that of the 2015–2019 average; on the contrary, for dental practitioners (g) a significant excess of mortality was still observed, equal to +227%.

The higher mortality observed among dentists can be explained in different ways: the increased risk associated with the way of practicing dentistry, or the fact that the group of physicians includes both those who carry out high-risk activities (for example, family medicine, emergency medicine, etc.) and doctors who, on the other hand, can be considered at lower risk and that this may have moderated the statistical effect. Therefore, a limitation of this study is that it has not been able to classify physicians according to the specialty practiced.

From the analysis of the standardized mortality rate compared to the population of the province of Pavia, it emerges that doctors had a significantly lower mortality rate than the local population both in the years preceding the pandemic and in 2020 and 2021. Therefore, although it is possible to state that the increase in mortality among healthcare professionals was particularly high compared to the general population and that a significant increase in mortality was observed compared to previous years, the COVID-19 epidemic produced a higher mortality in the general population, standardized by age, than healthcare professionals, and that this higher mortality is continuous over time even in the years preceding the pandemic. Therefore, these data could indicate that in healthcare professionals, although the increase in mortality was significantly high, other pre-existing factors made it impossible to achieve the high mortality rate of the population. These factors can be related to lifestyle and lower comorbidity linked to other pathologies. Table 4 shows that the SMR of all dentists was higher in 2018; however, in that year the COVID-19 epidemic had not yet broken out. This result must be carefully considered because it could be interpreted incorrectly: it does not indicate that dentist mortality was higher in 2018 (dentist mortality was higher in 2020, Table 2); this result answers the question as to whether the mortality of dentists was different from that of the resident population in 2018; the result clearly indicates that even in 2018 the mortality of dentists was lower than that of the general population.

This study has some limitations: since this study only assessed the mortality rate, it is not possible to obtain conclusive indications on the causes of the changes in the mortality rate. Therefore, the reduction in the mortality rate related to doctors (d) observed in 2021 cannot be attributed with certainty to vaccination coverage, nor is it possible to quantify the effects of vaccination coverage on mortality.

Furthermore, during the pandemic the causes of mortality have changed compared to the previous 5 years, creating a scenario of causality that is difficult to interpret [35,51,57,58,59,60,61,62,63,64].

In addition, this study evaluated the data relating to a specific area, located in the north of Italy, in which the pandemic caused a very marked increase in mortality, among the worst in the world. The peculiar mortality in this area may have been influenced by different factors, such as the high population density, the high average age of the population, and governments policies. Therefore, the results and conclusions of this study are not valid for different scenarios and do not reflect the global impact.

This study can be useful because it provides data on the impact of an infectious health emergency (COVID-19) on the health personnel who operate in the front line on the care of the population; therefore, these data can support health policy decisions that consider the effects of a pandemic; moreover, these data are particularly useful because they relate to a territory where the pandemic had more serious effects.

## 5. Conclusions

This is the first study to retrospectively evaluate mortality among physicians and dentists in an area considered at high risk for the COVID-19 epidemic. There was a significant increase in mortality during the COVID-19 epidemic among doctors and dentists, and this increase was greater than in the general population. Dentists had a higher mortality rate than physicians as a whole, but this does not mean that physicians practicing a particular specialty (emergency medicine, family medicine, etc.) were not exposed to an even greater risk. There was a sharp decline in mortality among doctors in 2021, probably related to early and comprehensive vaccination coverage. This study allows us to understand the serious impact on mortality of the SARS-CoV-2 epidemic on medical workers and dentists operating in a high-risk area (Northern Italy). These data are important both retrospectively for scholars who seek to analyze the evolution of the pandemic, and prospectively to make appropriate health policy decisions in the future.

## Figures and Tables

**Table 1:** Groups analyzed in the study.

	Group	Description	Annotations
a	Italian population	Entire Italian population, without exclusion of sex or age groups	
b	Population of the province of Pavia	Entire population residing in the Province of Pavia, without exclusion of sex or age groups	
c	All those enrolled in the register of physicians and dentists	All those enrolled in the Province of Pavia register of physicians and dentists with any qualification	c = d + e + f = d + g
d	Physicians	Those enrolled in the Province of Pavia register only with the qualification of physician	
e	Dentists	Those enrolled in the Province of Pavia register only with the qualification of dentist	
f	Physicians-Dentists	Those enrolled in the Province of Pavia register with the qualification of both physician and dentist	f ≠ d + e
g	Total Dentists	Those enrolled in the Province of Pavia register with the qualification of both physician and dentist plus those enrolled in the register with only the qualification of dentist	g = e + f

**Table 2:** Death-related data on the Italian population, the resident population of the province of Pavia, and those enrolled in the provincial registry of physicians and dentists.

	2015	2016	2017	2018	2019	2020	2021	2015–2019 **
**Italy (a)**								
Dead	656,196	627,071	659,473	640,843	644,515	746,146	709,035	645,620 ± 12,960
Population on January 1st	60,295,497	60,163,712	60,066,734	59,937,769	59,816,673	59,641,488	59,236,213	60,056,077 ± 187,304
Mortality	1.09%	1.04%	1.10%	1.07%	1.08%	1.25%	1.20%	1.08 ± 0.02%
Mortality variation *	1.23%	−3.05%	2.13%	−0.54%	0.23%	16.37%	11.34%	
**Province of Pavia (b)**								
Dead	7129	6762	7196	6901	7008	9293	7352	6999 ± 174
Population	544,841	543,875	543,138	541,617	541,717	540,376	535,801	543,038 ± 1390
Mortality	1.31%	1.24%	1.32%	1.27%	1.29%	1.72%	1.37%	1.29 ± 0.03%
Mortality variation *	1.52%	−3.54%	2.79%	−1.14%	0.37%	33.43%	6.46%	
**All Doctors (c)**								
Dead	23	18	28	30	30	45	24	26 ± 5
Population	5100	5222	5282	5342	5394	5454	5529	5268 ± 114
Mortality	0.45%	0.34%	0.53%	0.56%	0.56%	0.83%	0.43%	0.49 ± 0.09%
Mortality variation *	−7.72%	−29.47%	8.47%	14.91%	13.80%	68.83%	−11.18%	
**Physicians (d)**								
Dead	21	16	26	24	26	36	17	23 ± 4
Population	4389	4513	4567	4622	4660	4744	4836	4550 ± 106
Mortality	0.48%	0.35%	0.57%	0.52%	0.56%	0.76%	0.35%	0.50 ± 0.09%
Mortality variation *	−3.51%	−28.51%	14.80%	4.71%	12.51%	53.03%	−29.11%	
**Dentists (e)**								
Dead	0	0	0	0	1	0	1	0 ± 0.45
Population	254	261	276	289	308	304	313	278 ± 22
Mortality	0.00%	0.00%	0.00%	0.00%	0.32%	0.00%	0.32%	0.06 ± 0.15%
Mortality variation *	−100.00%	−100.00%	−100.00%	−100.00%	400.00%	−100.00%	392.01%	
**Physicians-Dentists (f)**								
Dead	2	2	2	6	3	9	6	3 ± 2
Population	457	448	439	431	426	406	380	440 ± 13
Mortality	0.44%	0.45%	0.46%	1.39%	0.70%	2.22%	1.58%	0.69 ± 0.41%
Mortality variation *	−36.32%	−35.04%	−33.70%	102.58%	2.48%	222.58%	129.77%	
**All Dentists (g)**								
Dead	2	2	2	**6**	4	9	7	3 ± 2
Population	711	709	715	720	734	710	693	718 ± 10
Mortality	0.28%	0.28%	0.28%	0.83%	0.54%	1.27%	1.01%	0.44 ± 0.25%
Mortality variation *	−36.69%	−36.51%	−37.04%	87.57%	22.66%	185.32%	127.36%	

* Mortality variation with respect to the 2015–2019 arithmetic mean. ** Data reported in this column are arithmetic means of the 2015–2019 period ± standard deviation.

**Table 3:** Mortality rates 2020 and 21 vs. 2015–2019 average.

2020	All Doctors (c)	Physicians (d)	Dentists (e)	Doctors-Dentists (f)	All Dentists (g)
Observed mortality rate	0.83%	0.76%	0.00%	2.22%	1.23%
Expected mortality rate	0.49%	0.50%	0.07%	0.68%	0.45%
Number of deaths expected	27	24	0	3	3
Excess of mortality	0.34%	0.26%	−0.07%	1.54%	0.78%
Excess of deaths	18	12	0	6	6
MR% (95% CI)	**168 (131–206)**	**153 (112–193)**	0 (0–419)	**325 (207–443)**	**275 (167–383)**
Probability according to the Poisson distribution	***	**	N.S.	**	**
**2021**					
Observed mortality rate	0.43%	0.35%	0.32%	1.58%	1.01%
Expected mortality rate	0.49%	0.50%	0.07%	0.68%	0.45%
Number of deaths expected	27	24	0	3	3
Excess of mortality	−0.06%	−0.15%	0.25%	0.90%	0.56%
Excess of deaths	−3	−7	1	3	4
MR (95% CI)	89 (51–126)	71 (31–111)	443 (31–856)	**232 (110–353)**	**227 (115–338)**
Probability according to the Poisson distribution	N.S.	N.S.	N.S.	N.S.	*

Statistically significant data are in bold. N.S.: not significant; *: *p* < 0.05; **: *p* < 0.01; ***: *p* < 0.001.

**Table 4:** SMR% (CI95%). Registered doctors vs. general population of the province of Pavia.

	2015	2016	2017	2018	2019	2020	2021
All Doctors (c)	**17 (0–33)**	**13 (0–29)**	**18 (2–34)**	**19 (3–34)**	**18 (3–33)**	**20 (7–33)**	**12 (0–26)**
Physicians (d)	**18 (0–36)**	**14 (0–32)**	**20 (2–37)**	**18 (1–35)**	**19 (2–35)**	**19 (5–33)**	**10 (0–25)**
Dentists (e)	0 (0–122)	0 (0–114)	0 (0–109)	0 (0–104)	25 (0–123)	0 (0–86)	19 (0–105)
Physicians-Dentists (f)	**12 (0–59)**	**11 (0–56)**	**11 (0–56)**	**30 (0–74)**	**14 (0–56)**	**27 (0–63)**	**24 (0–63)**
All Dentists (g)	**10 (0–55)**	**9 (0–52)**	**9 (0–51)**	**25 (0–66)**	**16 (0–54)**	**23 (0–56)**	**23 (0–59)**

Statistically significant data are in bold.

## Data Availability

Publicly available datasets were analyzed in this study. This data can be found here: https://ordinemedicipavia.it/bollettino-ordine-dei-medici-pavia/, (accessed on 15 April 2022).
